# Annual Removal of Aboveground Plant Biomass Alters Soil Microbial Responses to Warming

**DOI:** 10.1128/mBio.00976-16

**Published:** 2016-09-27

**Authors:** Kai Xue, Mengting M. Yuan, Jianping Xie, Dejun Li, Yujia Qin, Lauren E. Hale, Liyou Wu, Ye Deng, Zhili He, Joy D. Van Nostrand, Yiqi Luo, James M. Tiedje, Jizhong Zhou

**Affiliations:** a State Key Joint Laboratory of Environment Simulation and Pollution Control, School of Environment, Tsinghua University, Beijing, China; b Institute for Environmental Genomics, University of Oklahoma, Norman, Oklahoma, USA; c Department of Microbiology and Plant Biology, University of Oklahoma, Norman, Oklahoma, USA; d School of Mineral Processing and Bioengineering, Central South University, Changsha, Hunan, China; e Research Center for Eco-Environmental Sciences, Chinese Academy of Sciences, Beijing, China; f Center for Microbial Ecology, Michigan State University, East Lansing, Michigan, USA; g School of Civil Engineering and Environmental Sciences, University of Oklahoma, Norman, Oklahoma, USA; h Earth and Environmental Sciences Division, Lawrence Berkeley National Laboratory, Berkeley, California, USA

## Abstract

Clipping (i.e., harvesting aboveground plant biomass) is common in agriculture and for bioenergy production. However, microbial responses to clipping in the context of climate warming are poorly understood. We investigated the interactive effects of grassland warming and clipping on soil properties and plant and microbial communities, in particular, on microbial functional genes. Clipping alone did not change the plant biomass production, but warming and clipping combined increased the C_4_ peak biomass by 47% and belowground net primary production by 110%. Clipping alone and in combination with warming decreased the soil carbon input from litter by 81% and 75%, respectively. With less carbon input, the abundances of genes involved in degrading relatively recalcitrant carbon increased by 38% to 137% in response to either clipping or the combined treatment, which could weaken long-term soil carbon stability and trigger positive feedback with respect to warming. Clipping alone also increased the abundance of genes for nitrogen fixation, mineralization, and denitrification by 32% to 39%. Such potentially stimulated nitrogen fixation could help compensate for the 20% decline in soil ammonium levels caused by clipping alone and could contribute to unchanged plant biomass levels. Moreover, clipping tended to interact antagonistically with warming, especially with respect to effects on nitrogen cycling genes, demonstrating that single-factor studies cannot predict multifactorial changes. These results revealed that clipping alone or in combination with warming altered soil and plant properties as well as the abundance and structure of soil microbial functional genes. Aboveground biomass removal for biofuel production needs to be reconsidered, as the long-term soil carbon stability may be weakened.

## IMPORTANCE

Global change involves simultaneous alterations, including those caused by climate warming and land management practices (e.g., clipping). Data on the interactive effects of warming and clipping on ecosystems remain elusive, particularly in microbial ecology. This study found that clipping alters microbial responses to warming and demonstrated the effects of antagonistic interactions between clipping and warming on microbial functional genes. Clipping alone or combined with warming enriched genes degrading relatively recalcitrant carbon, likely reflecting the decreased quantity of soil carbon input from litter, which could weaken long-term soil C stability and trigger positive warming feedback. These results have important implications in assessing and predicting the consequences of global climate change and indicate that the removal of aboveground biomass for biofuel production may need to be reconsidered.

## INTRODUCTION

Multiple biotic and abiotic factors in ecosystems are simultaneously altered with global change (1). On account of the rising concentrations of CO_2_ and other greenhouse gases (GHGs), the Earth’s surface temperature increased by 0.85°C from 1880 to 2012 and is expected to increase another 0.3 to 4.8°C in this century (2). Fossil fuel combustion and land use change have been the top two sources of CO_2_ emission and are vital for the soil carbon (C) budget (3, 4). Aboveground plant biomass removal (clipping) is a common land use practice in agriculture (5), animal grazing surrogate (6), natural grassland restoration (7), and the emerging biofuel industry. In the future, it is likely that warming and clipping will occur simultaneously, especially when current, routine biomass harvests expand to broader landscapes to facilitate bioenergy crop production.

Although realistic scenarios are almost certainly multifactorial (8), most global change studies associated with warming or clipping have focused on only a single factor. Influences of multiple factors have primarily been addressed in some plant-based or soil geochemistry studies (1, 8–13) but relatively less in soil microbial community investigations (14–18). Ecosystem responses to multiple factors could be predicted based on single-factor studies where the influences of multiple factors are additive (no interaction) but not where synergistic (the observed effect [OE] of combined treatment is greater than the predicted effect [PE] based on treatments assessed independently) or antagonistic (the observed effect is smaller than the predicted effect) interactions occur (1). Thus, multifactorial experiments are required to understand ecosystem responses, including microbial responses, to multiple factors (19).

Separately, warming and clipping alter soil and plant properties in either similar or contrasting fashions. For example, in grasslands, both warming and clipping were observed to increase soil temperature and decrease soil moisture (20). In contrast, warming increased net primary productivity (NPP) and plant C input to soil (21), but clipping reduced both (22). Moreover, warming led to an extended growing season length (22), while clipping caused compensatory root growth and stimulated C exudation (22, 23). The interactive effects of warming and clipping on these soil and plant properties rely on the mechanisms governing each single factor and the degree to which these factors interact (1); the latter is poorly understood. The responses of soil microbial communities to two or more factors are even less predictable (14, 15) than those of soil and plant properties, owing to their extremely high diversity, to the comparative lack of knowledge of functions, and to limitations in observations and data acquisition.

A single effect of clipping or warming on soil microbial communities has been investigated in many studies. Clipping was observed to decrease soil microbial biomass measured by fumigation-extraction (18), to reduce microbial activity and diversity tested by BIOLOG (24), and to shift microbial community structure, e.g., to a decreased fungus/bacterium ratio (25, 26) or to increased numbers of Gram-positive bacteria in comparison to saprophytic fungi as determined by levels of phospholipid fatty acids (PLFAs) (27). However, clipping did not change the soil bacterial community structure investigated by sequencing 16S rRNA genes in semiarid grassland soils, either alone or together with multiple environmental changes of plant invasion and nitrogen (N) fertilization (28). The influence of climate warming on compositions of soil microbial communities was evident by fingerprinting, taxonomic, and phylogenetic analysis in many studies (29–33). Recently, three primary feedback mechanisms mediated by microorganisms were discovered to regulate soil C dynamics under warming conditions. These included the shift in microbial community composition that reduced the temperature sensitivity (indicated by Q_10_) of heterotrophic soil respiration, stimulation of genes encoding products that degrade labile but not recalcitrant C, and enhancement of nutrient-cycling genes (31). However, it is unclear whether these mechanisms would be affected by the simultaneous existence of warming and clipping. Thus, the long-term responses of soil microbial communities, in particular with respect to their functional potentials, to multifactorial effects of warming and clipping are investigated here.

This study evaluated the effects of 8 years of warming (averaging an ambient temperature of +2°C) and clipping on soil properties (e.g., soil temperature, moisture, bulk density, and C and N pools), plant properties (e.g., litter, biomass, and primary production), and soil microbial communities in a tall grass prairie ecosystem. Specifically, we evaluated the effects of clipping and warming on soil microbial communities by several approaches, including analysis of PLFA levels for microbial biomass, soil respiration for microbial community activity, and extracellular enzyme (phenol oxidase) activity, 454 sequencing of 16S rRNA genes for the phylogenetic composition of bacteria, and GeoChip for microbial functional genes. We focused on the GeoChip data as we found GeoChip to be the most sensitive method among the technologies adopted in this study. We hypothesized that warming and clipping would differentially affect abundances of functional genes in soil microbial communities and that these effects would be additive with respect to genes involved in C and N cycling. Specifically, enrichment of functional genes involved in recalcitrant C degradation was hypothesized to increase with clipping, responding to reduced fresh C input, but not with warming. Enrichment of nitrogen cycling genes was expected to increase with warming, owing to enhanced nutrient cycling, but not with clipping.

## RESULTS

### Soil and plant properties.

Substantial impacts of treatments on soil and plant properties were observed (Table 1). Warming alone and in combination with clipping significantly (*P* ≤ 0.01) increased the soil temperature by 8.1% and 14.5%, respectively. Warming alone and the combined treatment decreased the soil moisture significantly (*P* ≤ 0.05) by 4.6% and 4.4%, respectively.

**TABLE 1:** Observed percent changes of soil and plant properties in response to warming or clipping alone, or their combination, the predicted additive effect of warming and clipping, and the types of the interaction

Category	Parameter	Parameter value or *P* value						Type of interactive effect^e^
		Yr	W^a^	C^b^	OE^c^	PE^d^	OE-PE	
Soil properties	Temperature	2007	**8**.13***	0.84	**14**.46***	8.97	**5**.49***	**Synergistic**
	Moisture	2007	**−4**.62**	−0.98	**−4**.42**	−5.60	1.18	Additive
	Bulk density	2005	2.17	**9**.04**	4.31	11.21	−6.90	Additive
	Total organic C	2008	4.05	−1.21	7.48	2.84	4.64	Additive
	Labile C Pool 1	2008	−0.69	−7.44	−1.86	−8.13	6.27	Additive
	Labile C pool 2	2008	12.90	−2.21	13.32	10.69	2.63	Additive
	Recalcitrant C pool	2008	1.11	1.77	7.97	2.88	5.09	Additive
	C derived from C_4_ plant	2008	**18**.81**	4.36	**22**.26***	23.17	−0.91	Additive
	Total N	2008	0.79	−3.23	5.01	−2.44	7.45	Additive
	NH_4_^+^	2007	−6.56	**−19**.84***	1.40	−26.40	**27**.80***	**Synergistic**
	NO_3_^−^	2007	−23.76	−30.98	−34.57	−54.74	20.17	Additive
	C/N ratio	2008	2.80	1.47	0.34	4.27	−3.93	Additive
	^13^C	2008	**6**.61**	2.14	**8**.24***	8.74	0.50	Additive
	^15^N	2008	16.37	−3.43	**46**.07**	12.94	**33**.13***	**Synergistic**
	Phenol oxidase	2008	3.49	**16**.28**	39.71	19.77	19.94	Additive
								
Soil respiration	Total	2007	**29**.06**	19.84*	**52**.57**	48.90	3.67	Additive
								
PLFAs	Total PLFA	2008	**34**.91**	17.07	**55**.47**	51.98	3.49	Additive
	Fungi/bacteria	2008	−6.92	−13.84	−0.82	−20.76	19.94	Additive
								
Plant properties	C_3_ peak biomass	2007	34.13	35.15	50.55	69.28	−18.73	Additive
	C_4_ peak biomass	2007	**25**.05**	1.37	46.53*	26.42	20.11	Additive
	BNPP	2007	22.44	10.42	**109**.54***	32.86	**76**.68***	**Synergistic**
	Litter	2006	27.83	**−80**.80***	**−75**.27***	−52.97	−22.30	Additive

a W, warming effect alone, calculated as 100% × (UW − UU)/UU, where UW and UU represent the averaged values in unclipped-warmed and unclipped-unwarmed plots, respectively.

b C, clipping effect alone, calculated as 100% × (CU − UU)/UU, where CU represents the averaged values in clipped-unwarmed plots. The significance data are in bold font and labeled with *** where *P* = ≤0.01, ** where *P* = ≤0.05, and * where *P* = ≤0.10. The two-tailed paired *t* test was used for most variables, while the two-tailed permutation paired *t* test was used for C/N ratio and C_4_ peak biomass calculations.

c OE, observed effect, calculated as 100% × (CW − UU)/UU, where CW represents the averaged values in clipped-warmed plots.

d PE, predicted additive effect, calculated as [100% × (UW − UU)/UU +100% × (CU − UU)/UU].

e Interactive effect is additive when PE does not differ significantly from OE, synergistic when PE is significantly smaller than OE, or antagonistic when PE is significantly larger than OE.

Measured soil C pools (total organic C [TOC], labile C, and recalcitrant C) did not differ significantly under any of the treatment conditions. However, based on ^13^C data, the proportion of soil C derived from C_4_ plants significantly (*P* ≤ 0.01) increased by 18.8% and 22.3% in response to warming alone and the combined treatment, respectively. For N, the soil NH_4_^+^ content significantly (*P* = 0.004) decreased by 19.8% in response to clipping alone. The soil ^15^N content significantly (*P* = 0.01) increased by 46.1% with the combined treatment. The levels of total N or NO_3_^−^ availability or soil C/N ratios did not differ significantly under any treatments.

Clipping alone and the combined treatment significantly (*P* ≤ 0.01) decreased the litter mass by 80.8% and 75.3%, respectively, given that yearly aboveground biomass (AGB) removal in clipped plots (100.4 g C m^−2^ year^−1^ on average) accounts for 46.4% of the aboveground net primary production (ANPP) (21). As observed previously (31), warming alone increased the C_4_ peak biomass significantly (*P* = 0.01) but not the C_3_ peak biomass. The combined effects of warming and clipping also increased the C_4_ peak biomass at a marginally significant level (*P* = 0.058) as shown by the two-tailed permutation paired *t* test. In addition, the belowground net primary production (BNPP) significantly (*P* = 0.003) increased with the combined treatment by 109.5%.

### Soil microbial community.

Levels of both total PLFAs representing soil microbial community size and soil respiration reflecting microbial community activity increased significantly (*P* ≤ 0.05) with warming alone and the combined treatment (Table 1). The microbial functional composition measured by GeoChip was altered significantly (*P* ≤ 0.05) by warming alone and clipping alone, as well as by the combined treatment (Table 2). However, the microbial compositions resulting from these three treatments assessed by 454 sequencing of 16S rRNA genes differed only at marginally significant levels (*P* ≤ 0.10), and PLFA compositions differed significantly (*P* = 0.012) only with the combined treatment. In detrended correspondence analysis (DCA) profiles, the treatment data corresponding to both the GeoChip and the 16S rRNA gene communities were separated well, but no treatment effect was observed for the PLFA compositions (see Fig. S4 in the supplemental material). Regarding community alpha diversity, clipping alone increased the community richness, Shannon, Simpson, and inverse Simpson diversity indices at marginally significant levels (*P* ≤ 0.10) based on GeoChip data, while it decreased the inverse Simpson diversity index based on the 16S rRNA gene data (see Table S4). At the phylum level, in analysis of 16S rRNA genes, only the levels of *Actinobacteria* and Op10 sequences increased with warming alone at marginally significant levels (*P* ≤ 0.10), while the levels of *Planctomycetes* deceased with warming alone at a marginally significant level (*P* ≤ 0.10) (see Fig. S5).

**TABLE 2:** Pairwise dissimilarities tested by permutational multivariate analysis of variance (Adonis) for GeoChip-detected functional genes, 454 sequencing of 16S rRNA genes, and PLFA, all based on Horn dissimilarity distance^a^

Comparison	GeoChip		454 sequencing		PLFA	
	*F*	Pr(>*F*)	*F*	Pr(>*F*)	*F*	Pr(>*F*)
UW versus UU	2.104	**0.032**	2.055	0.077	0.349	0.970
CU versus UU	2.469	**0.002**	2.597	0.060	0.115	0.704
CW versus UU	1.952	**0.027**	1.970	0.095	26.09	**0.012**

a UU, unclipped-unwarmed communities; UW, unclipped-warmed communities; CU, clipped-unwarmed communities; CW, clipped-warmed communities. Bold values represent significance at *P* ≤ 0.05. F, F-test statistics; Pr(>F), p value, the probability of obtaining a larger F value from permutations than the F value based on actually observed data.

**FIG 1:**
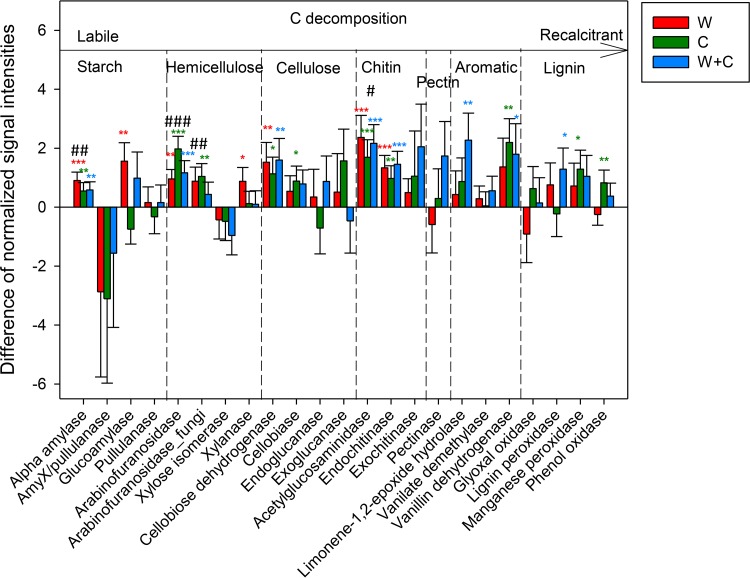
Signal changes of detected C degradation genes measured by GeoChip in response to treatments. Bars presented show means ± standard errors (*n* = 6 × gene probe number) of the signal intensities of the detected carbon degradation genes in response to warming alone (W), clipping alone (C), and their combination (W + C) compared with the control (unclipped-unwarmed treatment). Significance of treatment effect is indicated by *** where *P* = ≤0.01, ** where *P* = ≤0.05, and * where *P* = ≤0.10 and in the same colors as the bars for the different treatment groups. Significance of interaction between warming and clipping is labeled with ### where *P* = ≤0.01, ## where *P* = ≤0.05, and # where *P* = ≤0.10 (see Table S2 in the supplemental material).

More importantly, different groups of functional genes involved in C degradation measured by GeoChip were differentially affected by warming and clipping. Warming alone increased the abundance of functional genes only for degrading labile C at significant or marginally significant levels (*P* ≤ 0.10), while clipping alone increased the abundance of genes for degrading both labile and recalcitrant C at significant or marginally significant levels (*P* ≤ 0.10), including those encoding vanillin dehydrogenase for aromatic component degradation (by 122%), as well as those encoding manganese peroxidase (by 47.0%) and phenol oxidase (by 53.9%), which are involved in lignin degradation (Fig. 1). Consistently, the enzyme activity of phenol oxidase significantly (*P* = 0.03) increased by 16.3% in response to clipping alone (Table 1) but not in response to warming alone. Moreover, the combined treatment increased the abundance of genes degrading both labile and recalcitrant C at significant or marginally significant levels (*P* ≤ 0.10) (Fig. 1).

**FIG 2:**
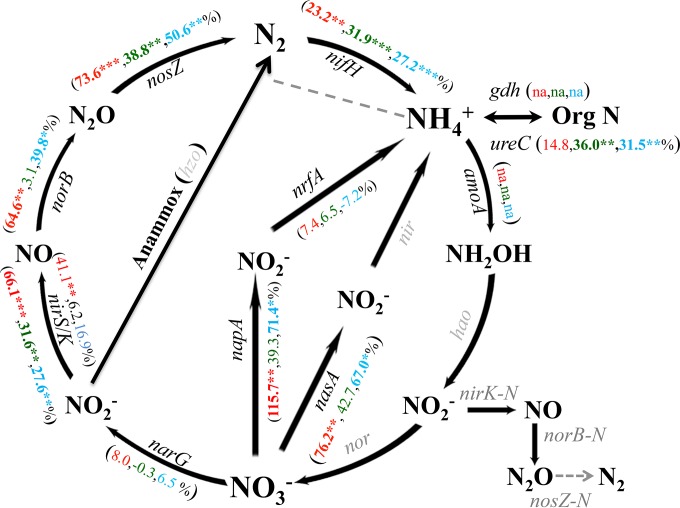
Percentage signal changes of detected N cycling genes measured by GeoChip in response to treatments. The numbers corresponding to each gene represent the signal changes in percentage induced by warming alone (dark red), clipping alone (orange), and their combined treatment (yellow green) compared with the control (unclipped-unwarmed treatment) (*n* = 6 × gene probe number). Significance is indicated by *** where *P* = ≤0.01, ** where *P* = ≤0.05, and * where *P* = ≤0.10. Gray-colored genes are not represented in the version of GeoChip used in this study or were undetected. “na” means that the gene was not detected in control samples but was detected with the warming treatment alone, the clipping treatment alone, or the combined treatment.

Differential effects of warming and clipping on N-cycling genes were also observed (Fig. 2). Warming alone significantly (*P* ≤ 0.05) increased the levels of *nifH* associated with N_2_ fixation, *nasA* for assimilation, and almost all denitrification genes. Clipping alone significantly (*P* ≤ 0.05) increased the abundance of genes related to ammonium production, including *ureC* for ammonification (by 36.0%) and *nifH* (by 31.9%), and increased the abundance of denitrification genes *nirK* (by 31.6%) and *nosZ* (by 38.8%). Moreover, the combined treatment increased the abundance of almost all (7 of 10) detected genes involved in N cycling at significant or marginally significant levels (*P* ≤ 0.10), similarly to the warming effect alone.

Other than changes in functional genes, the estimated Q_10_ of soil respiration was significantly (*P* ≤ 0.05) decreased by warming and clipping alone, as well as by the combined treatment (see Fig. S1 in the supplemental material), suggesting reduced temperature sensitivity of microbial respiration in response to these treatments.

### Interactions between warming and clipping.

Additive interactions were observed as the main pattern for most soil and plant properties (e.g., soil total organic carbon and C_3_ peak biomass) (Table 1; see also Table S1 in the supplemental material). However, significant (*P* ≤ 0.05) synergistic interactions were observed for a few important variables, including soil temperature, NH_4_^+^ content, δ^15^N, and BNPP (Table 1). With respect to 454 sequencing of 16S rRNA genes, marginally significant (*P* ≤ 0.10) antagonistic and synergistic interactions were observed for *Actinobacteria* and *Planctomycetes*, respectively.

**FIG 3:**
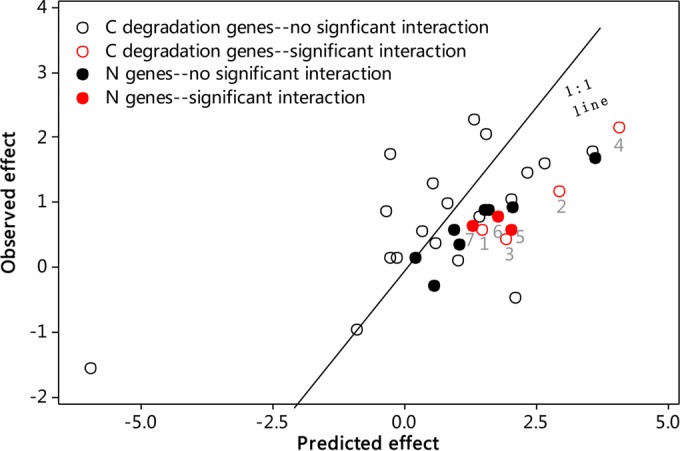
Predicted and observed effects of combined warming and clipping treatment on C degradation and N genes based on GeoChip measurements. C degradation genes are plotted with open circles and N genes with filled circles. The observed effects were calculated as the signal difference between $\overset{¯}{CW}$ and $\overset{¯}{UU}$ ($\overset{¯}{CW}-\overset{¯}{UU}$), where $\overset{¯}{CW}$ represents average gene signal intensity in clipped-warmed plots and $\overset{¯}{UU}$ represents that in unclipped-unwarmed plots. The predicted effects were the sum of individual effects of warming and clipping $\left[(\overset{¯}{UW}-\overset{¯}{UU})+(CU-\overset{¯}{UU})\right]$, where $\overset{¯}{UW}$ represents average gene signal intensity in unclipped-warmed plots and CU represents that in clipped-unwarmed plots. Points in 1:1 lines represent the additive responses to combined treatment (no interaction); points above and below the 1:1 line represent synergistic and antagonistic interactions, respectively. Significant interactions (*P* < 0.05) are shown in red for C degradation genes (1-*amyA*, 2-*ara*, 3-*ara_fungi*, 4-*acetylglucosaminidase*) and N cycling genes (5-*nirK*, 6-*nosZ*, 7-*nifH*). Error bars of predicted and observed effects were omitted for clarity. See Table S2 in the supplemental material for details.

In the context of C degradation genes, additive interactions were also a common pattern (see Table S2 and S3 in the supplemental material). However, antagonistic interactions were observed for genes encoding α-amylase (*amyA*) utilized for starch degradation (*P* = 0.03), arabinofuranosidase (*ara*) from either bacteria or fungi involved in hemicellulose degradation (*P* ≤ 0.05), and acetyl-glucosaminidase associated with chitin degradation (*P* = 0.06) (Fig. 1 and 3; see also Table S2 and S3).

Interactions between warming and clipping tended to be antagonistic for all N cycling genes (Fig. 3; see also Table S2 and S3 in the supplemental material). The antagonistic interactions were significant (*P* ≤ 0.05) for *nirK*, *nosZ*, and *nifH*. The antagonistic interactions on N cycling genes could also be observed by comparing the warming effects with and without clipping. Though warming increased the abundance of most N cycling genes significantly (*P* ≤ 0.05) without clipping, it did not change the abundance of any N cycling genes with clipping (see Table S3).

### Linkages between microbial communities with soil and plant properties.

At the whole-community level, canonical correspondence analysis (CCA) identified significant correlations (*P* ≤ 0.05) between microbial compositions based on either functional genes or 16S rRNA genes and a set of key soil and plant properties (see Fig. S2 and S3 in the supplemental material). The adopted model explained 63.6% of the variation in the functional gene compositions and 67.8% of the 16S rRNA gene variation. Partial CCA-based variation partitioning analysis (VPA) demonstrated that soil temperature and moisture explained 8.0% to 8.4% of the variation, whereas plant properties explained 25.4% to 26.1% and other soil properties explained 25.5% to 27.7%.

To further investigate the plant and soil properties related to shifts in the C and N cycling functional gene changes, Mantel tests were performed (see Table S5 in the supplemental material). The structures of all C degradation genes were significantly (*P* ≤ 0.05) correlated with soil temperature and variables closely linked to soil substrate, i.e., litter mass, BNPP, C_4_ species peak biomass, and soil recalcitrant C pool. Levels of lignin degradation genes were also significantly (*P* ≤ 0.05) correlated with soil NH_4_^+^ content and total N content. A matrix of all genes involved in N cycling showed a significant (*P* ≤ 0.05) correlation with soil NH_4_^+^ rather than NO_3_^−^ content. Significant correlations (*P* ≤ 0.05) between litter input or soil C components and the levels of certain N cycling genes were also observed.

## DISCUSSION

### Soil and plant properties.

At the long-term, multifactorial field experiment site, distinct changes of soil and plant properties in response to warming and clipping treatments were observed, which agrees with previous findings, especially with respect to the soil microclimate (e.g., temperature) and soil C input from plants (12, 20–22, 31, 34–36). The decreased litter mass and increased BNPP in response to clipping alone or in combination with warming revealed changes in the quantity of soil C inputs. Also, more soil C derived from C_4_ plants in response to warming alone and the combined treatment contributed to the decreased quality of soil C input. C_4_ plant materials had a higher C/N ratio than C_3_ plant materials as measured at the site in 2008 (35.0 for C_3_ and 63.0 for C_4_ leaf tissues, *P* ≤ 0.01) and likely greater lignin content (37).

Despite alterations in both the quantity and the quality of the soil C input, there were no significant changes in measured soil C pools. This was likely due to the offsetting effects of different components (e.g., soil C quantity and quality) in determining the soil C balance and/or the enormous soil C pool size (38) relative to the newly input fraction.

### Soil microbial communities.

Shifts in soil microbial functional gene composition with warming alone, clipping alone, and the combined treatment were observed by GeoChip analyses. The warming-induced shift in soil microbial community composition coincides with the general notion observed in this other experiment site (18, 31, 39, 40) and other experiment sites (33, 41–44). Conflicting results were obtained for clipping influences on soil community composition (24–28), which is likely a product of variations in the intensities of biomass removal, local niches, and/or the investigation methods.

We adopted PLFA analysis, 454 sequencing of the 16S rRNA genes, and GeoChip analysis to investigate microbial community compositions in this study. PLFA analysis and 454 sequencing were less sensitive than GeoChip analysis in detecting changes of microbial compositions. PLFA compositions were changed significantly only by the combined treatment, and only the abundances of the 16S rRNA gene communities were changed at marginally significant levels, while the microbial functional composition assessed by GeoChip consistently showed significant alterations under all treatment conditions. As individual PLFAs are not species specific, PLFA analysis cannot be used to represent the taxonomic diversity of microbial communities (45, 46). For microbial phylogenetic composition assessments by 454 sequencing, the lower sensitivity was likely due to the high noise associated with random sampling (47, 48). Random sampling is common in sequencing-based technologies and is particularly a problem when microbial communities are complex but sampling efforts (sequencing depth and coverage) are limited (47). In contrast, GeoChip is less susceptible to random sampling artifacts. Other than methodology issues, microbial functional genes might also be more responsive to treatments (ecological niches) than phylogenetic “species” (49). It is possible that ecological niches are colonized randomly by various species possessing similar functions (50, 51), but members of these within functionally equivalent groups may or may not be phylogenetically related. This is especially true in considering the relatively common occurrence of bacterial lateral gene transfer (49).

The temperature sensitivity of soil respiration has an important influence on the feedback between C cycling and global changes (31, 52, 53). The estimated Q_10_ significantly decreased with warming alone and clipping alone as well as with their combination (see Fig. S1 in the supplemental material). Previously, reduced temperature sensitivity of soil respiration under conditions of warming alone was proposed as a negative-feedback mechanism resulting from the adaptive changes in the soil microbial community composition, rather than from soil substrate depletion, as evidence showed that substrates were not depleted (31). However, in this study, the harvest of aboveground plant biomass under conditions of both clipping alone and the combined treatment may have led to a soil substrate deficiency for soil microorganisms. Thus, it is hard to discern whether the reduced Q_10_ level seen with clipping alone or the combined treatment was driven by changes in soil C substrates or in soil microbial community shifts or both. Thus, it is still unclear whether or not the decreased temperature sensitivity could diminish the positive feedback between C cycling and global changes.

### Carbon degradation genes.

Abundances of functional genes encoding enzymes involved in the degradation of relatively recalcitrant C substrates increased with clipping alone and with the combined treatment, indicating declines in the levels of fresh and labile C substrates. The removal of harvested biomass under conditions of both clipping alone and the combined treatment likely decreased the levels of fresh and labile C input into soils as implied by the substantially reduced litter mass levels and hence increased the proportion of old, recalcitrant C in the soil C pool. As noted previously, a soil microbial community may function differently in various scenarios; e.g., high temperatures induced the metabolism of substrates that were not utilized in low temperatures (33). Thus, a higher proportion of recalcitrant C in a soil C pool might drive an increase of the abundance of organisms harboring genes useful for degrading recalcitrant C substrates. Consistently, as shown by the Mantel test, the structures of the C degradation genes in the communities were significantly correlated with litter mass, BNPP, C_4_ species peak biomass, and the soil recalcitrant C pool, all of which are important in determining the quantity and quality of soil substrates. Soil total N and available NH_4_^+^ contents were also correlated with levels of lignin degradation genes, likely indicating that N mediated or responded to the process of lignin degradation, supportive of previous findings (54, 55). Moreover, in the combined treatment, the increases in the abundance of recalcitrant C degradation genes suggested that increases in C_4_ peak biomass and BNPP could not completely compensate for the effect of litter mass loss in terms of available labile C for microbial decomposition.

Functional gene changes involved in C degradation may have substantial impacts on ecosystem functioning. In the same experiment site, enrichment of genes for labile but not recalcitrant C degradation in response to warming alone was proposed as a negative-feedback mechanism of ecosystem response to warming (31). In the dual-factorial context in this study, the increases in the abundance of genes for both labile and recalcitrant C degradation in response to clipping alone or the combined treatment suggested that the soil microbial community in these treatments had the potential for enhanced decomposition of C, including the recalcitrant fractions. Thus, the long-term stability of soil C storage may be interrupted, which would weaken microbially mediated negative feedback corresponding to ecosystem responses to warming (31). A previous study suggested that the stability of C in deep soil layers may be maintained because of the absence of fresh C input (56). Our results show that, even with less fresh C input owing to clipping, the stability of soil C may still decrease through the directional shift of the soil microbial community toward recalcitrant C decomposition.

### Nitrogen cycling genes.

Nitrogen is a common limiting factor for C cycling in terrestrial ecosystems (TECOs) and critical for ecosystem functions (57), e.g., sustaining plant growth. Increases in the abundance of most detected N cycling genes in the combined treatment were consistent with increased soil ^15^N, which suggested accelerated rates of N-cycling processes (58, 59).

Some specialized metabolic processes limited to relatively few species have been closely linked to the taxonomic or phylogenetic diversity of specific microbial groups, e.g., CH_4_ consumption and methanotroph richness (60), as well as nitrification potential and bacterial *amoA* abundance (61, 62). In contrast, soil processes broadly catalyzed by many microbes would be difficult or impractical to relate to the taxonomic or phylogenetic diversity of microbial communities (63), likely owing to functional redundancy (60) and/or the facultative nature of these processes (64). However, some of these broad processes were well linked to the same functional gene from different genera, e.g., denitrification (N_2_O emission) and denitrifying gene (*nirS* and *nosZ*) abundances (65) and the activity of enzymes directly linked to C degradation at field-to-regional scales and corresponding C degradation genes (66). In this study, we observed that the abundance of genes associated with ammonium production (*nifH* and *ureC*) increased under the clipping-alone treatment conditions. This preliminary finding insinuates that the decline in soil NH_4_^+^ levels with clipping may be compensated by stimulated microbial ammonium-producing processes. As a result, the plant peak biomass (either C_3_ or C_4_) and BNPP did not decrease under the conditions that included lower soil NH_4_^+^ levels. However, further investigations are needed to verify the linkage between these functional genes and N_2_ fixation rates and urease activity, as well as to determine whether the increased abundance of denitrification genes (e.g., *nirK*) would lead to higher levels of N_2_O emissions and whether increased levels of the nitrous-oxide reductase gene (i.e., *nosZ*) would reflect transformations of N_2_O to N_2_. This report provides a basis for such further investigations to determine the relative importance of these mechanisms in determining the overall direction of ecosystem functioning.

### Interactions between warming and clipping.

Other than additive interactions, effects of antagonistic interactions between warming and clipping on functional genes and of the synergistic interaction on soil temperature were observed in this study. Though effects of interactions of environmental factors on soil microbial community properties were involved in a few studies (14, 15, 17, 18), the direction of interactions was seldom investigated. In contrast, effects of additive (1, 67), antagonistic (8, 9, 68, 69), and synergistic (16, 70, 71) interactions on plant properties or soil biochemical processes were all reported. In a meta-analysis study focusing on soil respiration (72), the majority (90.6%) of interactions between environmental factors (elevated CO_2_, N addition, irrigation, and warming) were additive, while antagonistic and synergistic interactions were denoted to a much lesser extent. Consistently, additive interactions also represented the main pattern in this study for most soil and plant properties, as well as for C degradation genes. Moreover, the results corresponding to the effects of a synthesis of perturbations (i.e., warming, elevated CO_2_, and drought) on N cycling processes in the CLIMAITE climate change experiment showed that antagonistic interactions (e.g., warming and elevated CO_2_ or drought interactions affecting NH_4_ consumption and grass N mineralization) dominated the interactive effects (72). Similarly, the effects of interactions between warming and clipping on all N cycling functional genes in this study also tended to be antagonistic.

Results showing effects of antagonistic interactions between warming and clipping on functional genes but of synergistic interaction on soil temperature indicate that the direct effect operating through heating the soil might not be the main force driving gene changes. Instead, the indirect warming effects related to microbial substrate changes mediated by plants might be crucial. If the direct warming effect (soil temperature change) were dominant, one would expect to observe more gene changes in response to warming combined with clipping, as the warming-induced soil temperature increase was amplified from 1.22°C without clipping to 2.04°C with clipping. Nevertheless, our study showed an opposing result and antagonistic interactions affecting functional genes. Moreover, the VPA results revealed that the indirect effects of warming (through soil substrate and plant responses) accounted for more than 52% of the total variance in soil microbial community compositions, while soil temperature and moisture accounted for only 8%. The importance of indirect effects on the plant community and other ecosystem properties in this experiment site (73) and other experiment sites (14, 19, 74) was also observed. However, it is also possible that the microbial response to temperature increase is nonlinear, that a threshold had been passed, or that the temperature increase (from 1.22 to 2.04°C) was not enough to cause a discernible divergence in soil microbial communities in our system. In previous reports, 1 to 3°C soil temperature increases either affected (75, 76) or did not change (77, 78) the grassland microbial community structure, depending on the site locations, investigation methods, and other interacting factors.

### Broader implications.

Overall, this study demonstrated that the land use practice of clipping tends to interact antagonistically with warming to alter microbially mediated feedback responses. The microbially mediated changes observed in this study could be important for projecting future climate warming effects in a multifactorial context. Owing to the complexity of ecosystem responses to multiple factors, the theoretical foundation for models to predict consequences of multiple global changes is incomplete (8). Our results provide insights into how soil microbial communities potentially mediate C and N cycling processes in response to experimental warming and clipping. These results could help to formulate mechanistically based hypotheses to improve the accuracy of model predictions. In addition, the results described in this study may provide valuable information for policy makers in considering land use change and practice for biofuel production. Biofuels that are derived from low-input high-diversity (LIHD) mixtures of native grassland perennials are believed to have great GHG reduction potential and do not directly compete with food production (79). However, most evaluations of biofuels have relied only on direct GHG emissions and/or energy use and are too narrow in scope (79, 80). Our results showed that clipping in LIHD grasslands may result in a higher potential for recalcitrant C decomposition and affect ecosystem feedback with respect to climate warming. These phenomena illustrated the complexity of microbial responses to climate change in more-realistic scenarios and revealed that single-factor studies cannot predict all relevant changes in soil microbial communities.

## MATERIALS AND METHODS

### Site description.

This study was conducted in a tall grass prairie ecosystem in the United States Great Plains in central Oklahoma (34°59′N, 97°31′W), as described previously (21). The mean annual temperature from 1948 to 1999 was 16.3°C, while the mean annual precipitation was 967 mm (Oklahoma Climatological Survey). The soil is silt loam (36% sand, 55% silt, and 10% clay in the top 15 cm) and is part of the Nash-Lucien complex, typically having high fertility, neutral pH, high available water capacity, and a deep and moderately penetrable root zone (81).

The experiment was established in November 1999. A paired factorial design was applied in which warming (targeting ambient +2°C) was set as a main factor and clipping as a nested factor. The warming treatment was achieved by suspending infrared radiators (Kalglo Electronics, Bethlehem, PA, USA) at 1.5 m above the ground. Dummy infrared radiators were used in control plots to mimic the shading effect from the device. There were six pairs of unwarmed and warmed plots. Each unwarmed or warmed plot (2 m by 2 m) was divided into four 1-m-by-1-m subplots. Plants in two diagonal subplots were harvested annually at 10 cm above the ground, usually in August, as the clipping treatment.

### Field measurements and Q_10_ estimation.

Soil temperature was measured every 10 min by thermocouples installed at a 2.5-cm depth in subplot centers. All thermocouples were connected to a CR10 Datalogger (Campbell Scientific Inc., Logan, UT, USA). Data corresponding to average soil temperatures over 1 h were stored in an SM196 storage module (Campbell Scientific Inc., Logan, UT, USA). In addition, soil moisture (percent volumetric) within the top 15 cm was measured by Time Domain Reflectometry (Soilmoisture Equipment Corp., Santa Barbara, CA) twice a month. In this report, the yearly averaged data of soil temperature and moisture in 2007 are presented.

Soil respiration was measured once or twice a month with a Li-COR 6400 portable photosynthesis system attached to a soil CO_2_ flux chamber (Li-COR Inc., Lincoln, NE), as described previously (21). A deep polyvinyl chloride (PVC) tube (80 cm^2^ in area and 70 cm in depth) in each plot was used to measure heterotrophic respiration by preventing new root growth inside the tubes and thus excluding autotrophic respiration. The CO_2_ efflux measured above the PVC collars, each only 5 cm in depth (80 cm^2^ in area), represented total soil respiration, including both heterotrophic and autotrophic respiration. The respiration data presented in this study were from 2007.

We used the inverse analysis method (82) to estimate Q_10_ values for heterotrophic soil respiration. Briefly, Bayesian paradigm was used to incorporate *a priori* probabilistic density functions (PDF) with aboveground biomass and heterotrophic soil respiration measurements from 2002 to 2008 to generate *a posteriori* PDF for Q_10_ values for heterotrophic soil respiration. Seven parameters (heterotrophic soil respiration Q_10_, exit rates of C from foliage pool, fine root pool, little pool, fast soil organic matter [SOM], slow SOM, and passive SOM pools) were estimated by incorporating soil respiration, heterotrophic soil respiration, aboveground biomass, and daily C inputs into a revised terrestrial ecosystem (TECO) model (82).

The lower and higher limits of Q_10_ (1.5 and 5) were chosen based on previous studies of Q_10_ values performed at the same site using regression methods (53, 83). With Bayes’ theorem, the posterior PDF *p*(*c|Z*) is given by *p*(c | Z) ∝ p(*Z|c*) *p*(*c*)*.* The analysis of probabilistic inversion was conducted using a Metropolis-Hastings (M-H) algorithm to construct the posterior probability density function of parameters (84, 85). The M-H algorithm samples random variables in high-dimensional probability density functions in the parameter space via a sampling procedure based on Markov chain Monte Carlo (MCMC) theorems. We ran the TECO model with each proposed parameter, and then we compared the modeled data (soil respiration and biomass) with the observed data. We constructed the *a posteriori* PDF of heterotrophic Q_10_ based on the posterior distribution of Q_10_ obtained in the previous steps. The maximum likelihood estimates were identified by observing the parameter values corresponding to the peaks of their PDF.

### Plant growth and litter mass.

Aboveground biomass was measured in clipped plots directly by aboveground plant removal, and the harvested plants were separated into C_3_ and C_4_ species. Aboveground biomass in unclipped plots was indirectly estimated by pin-contact counts as described previously (36). Since there is no carryover of living biomass from previous years in this ecosystem, the peak AGB (in summer [July or August]) was considered to represent the ANPP. The root biomass was measured by collecting root materials in soil cores (5.2 cm in diameter and 45 cm in depth) taken from the field. Collected roots were oven dried at 65°C for 48 h. Based on the observed root biomass and previously reported root turnover rate, the BNPP was estimated as described previously (21). Both the ANPP and BNPP data presented in this report were from 2007.

The litter mass on the soil surface was collected from the field in April 2006, cleaned by soft brushes, and oven dried at 65°C to a constant weight. After weighing, litter materials were returned to the plots where they had been collected. Litter amounts in 2006 were expected to influence soil microbes sampled in 2007.

### Sampling and soil characteristics.

Within each of the six plots, soil samples were collected from four treatments (UU: no clipping-no warming; UW: warming-no clipping; CU: clipping-no warming; CW: clipping-warming), and each had six replicates.

A two-step acid hydrolysis procedure was adopted to determine soil C pools, as described previously (13). The top 15 cm of soil cores (2.5-cm diameter) collected in October 2008 were used for this analysis. Briefly, 500 mg of soil was sequentially hydrolyzed with 5 N and 26 N H_2_SO_4_ and the hydrolysates were collected as labile pool 1 (predominantly containing polysaccharides) and labile pool 2 (largely containing cellulose), respectively. The levels of soil total organic C (TOC) and labile C pools 1 and 2 were measured by the use of a Shimadzu TOC-5000A total organic carbon analyzer with an ASI-5000A Auto Sampler (Shimadzu Corporation, Kyoto, Japan) in the Stable Isotope/Soil Biology Laboratory at the University of Georgia (Athens, GA). The recalcitrant C pools were calculated by subtracting organic C in the labile pools (pools 1 and 2) from the soil TOC.

The soil *δ*^13^C and *δ*^15^N data were determined for samples collected in 2008 from the top 20 cm of the soil cores (4 cm diameter) at the University of Arkansas Stable Isotope Laboratory on a Finnigan Delta^+^ mass spectrometer (Finnigan MAT, Germany) coupled to a Carlo Erba elemental analyzer (NA1500 CHN combustion analyzer; Carlo Erba Strumentazione, Milan, Italy) via a Finnigan Conflo II interface. By using a two-compartment mixing model, the proportion of soil C derived from C_4_ species was calculated based on the *δ*^13^C values in soil and plant materials of C_3_ and C_4_ species, as described previously (86).

The soil NH_4_^+^ and NO_3_^−^ contents were extracted from soils collected in 2007 by the use of 1 M KCl and measured by the use of a Lachat Quickchem 8500 series 2 instrument (Lachat, Loveland, CO) in the Soil, Water and Forage Analytical Laboratory at Oklahoma State University (Stillwater, OK). The soil bulk density values from 2004 and 2005 were used in this study as an estimation of soil bulk density at sampling time.

### Microbial analyses.

Microbial analyses were performed for soil samples collected from the top 15 cm in April 2007 and October 2008. Each sample was composited from four soil cores (2.5-cm diameter) and sieved through 2-mm-pore-size sieves. All samples were transported to the laboratory immediately after sieving and stored at −80°C. Several metagenomic and conventional microbial analyses were performed, including (i) PLFA analysis (27) of 2008 samples to provide data corresponding to microbial community size and community composition; (ii) phenol oxidase activity analysis (87) for 2008 samples; (iii) functional gene array analysis (GeoChip 3.0) (88) for 2007 samples to examine the functional potential; and (iv) 16S rRNA gene-based targeted pyrosequencing (89) for 2007 samples to obtain phylogenetic information. The major focus of the manuscript is on GeoChip analysis.

### GeoChip analysis.

Soil DNA was extracted by freeze-grinding mechanical lysis as described previously (90) and purified using a low-melting-temperature agarose gel followed by a phenol extraction. The extracted DNA was used for both GeoChip and 454 pyrosequencing analyses.

GeoChip 3.0 was used to perform hybridization as described previously (88), including whole-community genome amplification (WCGA) with 50 ng template DNA, template labeling with Cy5 dye for 2.5 µg amplified DNAs, and hybridization. The labeled DNA was suspended in 120 µl hybridization solution containing 50% formamide, 3× SSC (1× SSC is 0.15 M NaCl plus 0.015 M sodium citrate), 10 µg of unlabeled herring sperm DNA (Promega, Madison, WI), and 0.1% SDS. After denaturation, hybridizations were performed with a Tecan HS4800 Pro hybridization station (Tecan, USA). After washing and drying were performed, microarrays were scanned by a ScanArray Express microarray scanner (PerkinElmer, Boston, MA) at 633 nm using a laser power of 90% and a photomultiplier tube (PMT) gain of 75%. ImaGene version 6.0 (Biodiscovery, El Segundo, CA) was then used to determine the probe signal intensities. A total of 5,537 functional genes were detected by GeoChip hybridization.

Raw signal intensities from ImaGene were submitted to the online microarray Data Manager (http://ieg.ou.edu/entrance.html) and analyzed by the following steps: (i) removing spots flagged as 1 or 3 by ImaGene that had a signal-to-noise ratio (SNR) of less than 2.0; (ii) normalizing signal intensities at three levels, in individual subgrids within a slide, in technical replicates, and across different slides; (iii) removing genes with the number of detected positive probes fewer than 33.3 of total designed probe number, and the genes with fewer than two detected positive probes; and (iv) removing probes appearing in only one replicate. After those steps were performed, a total of 3,022 functional genes were obtained. The sum of signal intensities in each sample was calculated, and the average of the sums was used to multiply the relative abundance of each probe. A natural logarithm transformation was performed for the amplified relative abundance plus 1. The raw and normalized GeoChip data can be accessed through the accession number GSE86527 in the GEO database.

### The 454 pyrosequencing analysis.

The 454 pyrosequencing analysis was performed for 16S rRNA genes. Briefly, PCR primers F515 (GTGCCAGCMGCCGCGG) and R907 (CCGTCAATTCMTTTRAGTTT) (91), targeting the V4–V5 hypervariable regions of bacterial 16S rRNA (*Escherichia coli* positions 515 to 907), were selected. A sample tagging approach was used (92), and 2 to 3 unique 6-mer tags were adopted for each sample. The tag was added to the 5′ end of both forward and reverse primers. The amplification mix contained 10 units of *Pfu* polymerase (BioVision, Mountain View, CA), 5 µl of *Pfu* reaction buffer, 200 µM deoxynucleoside triphosphates (dNTPs) (Amersham, Piscataway, NJ), and a 0.2 µM concentration of each primer in a volume of 50 µl. A 10-ng aliquot of genomic DNA was amplified with an initial denaturation at 94°C for 3 min, 30 cycles of 95°C for 30 s, 58°C for 60 s, and 72°C for 60 s, and a final 2-min extension at 72°C. The products from about 5 to 10 amplifications were pooled for each sample and purified by agarose gel electrophoresis. Amplicons of all samples were pooled in an equimolar concentration for 454 pyrosequencing (93) on an FLX 454 system (454 Life Sciences, Branford, CT). Both forward and reverse reads were recovered with an average length of around 240 bp. All pyrosequencing reads were initially processed using the RDP pyrosequencing pipeline (http://pyro.cme.msu.edu/) (91).

Amplicon sequencing data are subject to various artifacts (47, 48, 94). To minimize the impacts of such artifacts on the final data analysis, low-quality sequences were removed to minimize the effects of random sequencing errors, including (i) sequences that did not match the PCR primer at the beginning of a read; (ii) sequences with nonassigned tags; (iii) sequence reads <200 bp after the proximal PCR primer if they terminated before reaching the distal primer; and (iv) sequences containing more than one undetermined nucleotide “N.” Only the first 240 bp after the proximal PCR primer of each sequence were included. After that, the raw sequences were sorted and distinguished by unique sample tags. The tag and primers were then trimmed for each replicate. For all 56 tags, the number of sequence reads ranged from 889 to 4,352. A total of 98,022 effective sequences were obtained.

Sequences in all samples were aligned by RDP Infernal Aligner (95), and a complete linkage clustering method was used to define operational taxonomic units (OTUs) within a 0.03 difference (96). The singleton OTUs were removed, and the remaining sequences were sorted into each sample based on OTUs. The OTU sequences were then assigned to a taxonomy by the RDP classifier (97) with a confidence cutoff of 0.8. The lineage of each OTU was summarized with all phylogenetic information. OTUs appearing in only three or fewer tag replicates for each treatment were removed, resulting in 1,824 OTUs that were used for subsequent analysis. The relative abundance was then calculated. Sequenced samples from the same soil (biological sample) but with different tags were combined by averaging the relative abundance levels of each OTU from different tags.

### Phospholipid fatty acids.

Phospholipid fatty acids were extracted from 3.0 g of soil as described previously (27) and were analyzed by the use of a Hewlett-Packard Agilent 6890A gas chromatograph (Agilent Tech. Co., USA) equipped with an Agilent Ultra-2 (5% phenyl)-methylpolysiloxane capillary column (25 m by 0.2 mm by 0.33 mm) and flame ionization detector. The detected levels of PLFAs were notably low in sample 2UW, with many missing values compared to what was commonly observed in other samples. Hence, 2UW was excluded from all further data analyses. All PLFAs were used for estimating the total microbial biomass. The PLFAs selected to represent the bacterial biomass included a15:0, i15:0, 15:0, a17:0, cy17:0, i17:0, 17:0, 16:1ω5c, 16:1ω9c, and 18:1ω5c, while the fungal biomass was represented by 18:1ω9c (98, 99). Relative abundance data were used for PLFA community composition analysis.

### Extracellular enzyme activity.

Extracellular enzyme activity of phenol oxidase involved in lignin decomposition was analyzed as described previously (87). The enzyme was assayed spectrophotometrically using 3,4-dihydroxy-l-phenylalanine (L-DOPA) as the substrate, followed by quantification of a red oxidation product of L-DOPA. The activities were standardized using a commercial L-DOPA oxidase, mushroom tyrosinase (Sigma T3824). Triplicate analyses were performed for each sample and its control, for which the substrate solution was added upon completion of incubation.

### Statistical analyses.

All statistical analyses were performed using the Vegan package in R 2.9.1 (The R Foundation for Statistical Computing, Vienna, Austria) or the pipeline developed at the University of Oklahoma (http://ieg.ou.edu). The nonparametric multivariate analysis of variance (Adonis) was used to test the treatment effect on microbial community structure. Consistent results were obtained by Adonis using Bray-Curtis, Euclidean, or Horn dissimilarity distances, and the results that we present are based only on Horn dissimilarity distance. Detrended correspondence analysis (DCA) was adopted to visualize the treatment effect on microbial community structure. Microbial community alpha diversity was represented by richness, evenness, Shannon, Simpson, and inverse Simpson indices. Canonical correspondence analysis (CCA) and the Mantel test were performed to determine significant properties shaping the microbial community structure. A partial CCA-based variation partitioning analysis (VPA) was then performed to calculate the proportion of each property’s contribution to the community structure. Two-tailed paired *t* tests and analysis of variance (ANOVA) were employed to test treatment effects on most soil and plant properties. Two-tailed permutation paired *t* tests were employed to test treatment effects on gene abundances and alpha diversity indices, as well as on soil C/N ratios and C_4_ peak biomasses that did not pass the Shapiro-Wilk test for normality. We defined significance as α = 0.05 and marginal significance as α = 0.10.

To determine the direction (additive, synergistic, or antagonistic) of interactive effects of warming and clipping on soil and plant properties and on 16S rRNA and functional genes, we compared observed effects (OEs) and predicted additive effects (PEs) of the combined treatments (100). For each set of the soil and plant properties or functional gene signal intensities, OE was calculated as follows:

OE = 100% × (CW − UU)/UU

where CW and UU represent the averaged values for the CW and UU treatment groups, respectively. Similarly, PE was calculated as follows:

PE = 100% × (UW − UU)/UU +100% × (CU − UU)/UU

where UW, CU, and UU represent the averaged values for the corresponding treatment groups. The interactive effects are additive when OEs do not differ significantly from PEs, tested by two-tailed paired *t* tests or the two-tailed permutation paired *t* test. Interactive effects would be synergistic if OEs were significantly higher than PEs or antagonistic if OEs were significantly lower than PEs.

## SUPPLEMENTAL MATERIAL

Figure S1  The posterior distribution of modeled Q_10_ values for heterotrophic soil respiration. Download Figure S1, PDF file, 0.9 MB

Figure S2  Canonical correspondence analysis (CCA) for GeoChip data and soil and plant properties. Download Figure S2, PDF file, 0.1 MB

Figure S3  Canonical correspondence analysis (CCA) for 454 data and soil and plant properties. Download Figure S3, PDF file, 0.1 MB

Figure S4  Detrended correspondence analysis (DCA) for microbial community composition measured by GeoChip (a); 454 sequencing of 16S rRNA gene communities (b); and PLFAs (c). Download Figure S4, PDF file, 0.1 MB

Figure S5  Changes of 16S rRNA genes at the phylum level by 454 sequencing in response to treatments. Download Figure S5, PDF file, 0.1 MB

Table S1  Soil and plant properties (means ± standard errors) in different treatments and ANOVA results. Table S1, DOCX file, 0.02 MB

Table S2  Interactive effects of warming and clipping on carbon degradation and nitrogen cycling genes measured by GeoChip. Table S2, DOCX file, 0.02 MB

Table S3  Percent changes of carbon degradation and nitrogen cycling genes measured by GeoChip in response to warming in unclipped and clipped plots or in response to clipping in unwarmed and warmed plots. Table S3, DOCX file, 0.02 MB

Table S4  Changes of alpha diversity in response to treatments compared with the control results for functional gene compositions detected by GeoChip and for taxonomic compositions analyzed by 454 sequencing of 16S rRNA genes. Table S4, DOCX file, 0.02 MB

Table S5  Mantel tests for comparisons between individual C degradation and nitrogen cycling functional gene compositions based on GeoChip and the soil and plant property variables. Table S5, DOCX file, 0.02 MB
