# Supplementary material for: Annual Removal of Aboveground Plant Biomass Alters Soil Microbial Responses to Warming
Source: mBio. 2016 Sep 27;7(5):e00976-16. doi: 10.1128/mBio.00976-16 (PMC5040111; doi:10.1128/mBio.00976-16)
Supplement: Figure S1 — The posterior distribution of modeled Q10 values for heterotrophic soil respiration. Download [file mbo005163005sf1.pdf]

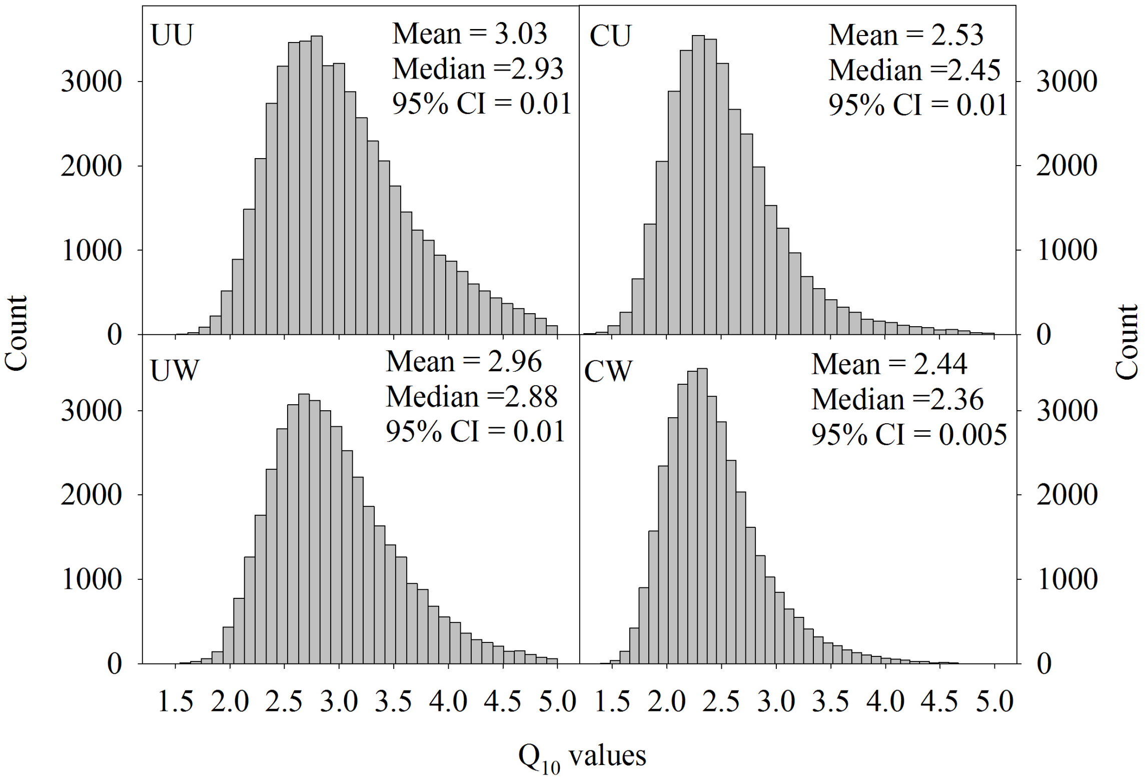

Fig. S1. The posterior distributions of modeled Q_{10} values for heterotrophic soil respiration. UU stands for unclipped-unwarmed, UW for unclipped-warmed, CU for clipped-unwarmed and CW for clipped-warmed plots.
