# Supplementary material for: Annual Removal of Aboveground Plant Biomass Alters Soil Microbial Responses to Warming
Source: mBio. 2016 Sep 27;7(5):e00976-16. doi: 10.1128/mBio.00976-16 (PMC5040111; doi:10.1128/mBio.00976-16)
Supplement: Figure S3 — Canonical correspondence analysis (CCA) for 454 data and soil and plant properties. Download [file mbo005163005sf3.pdf]

(A)

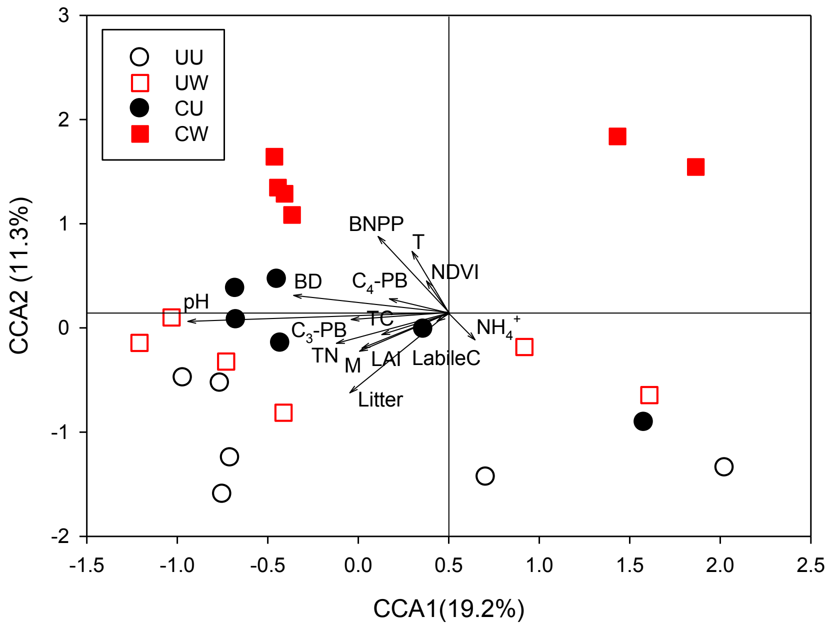


(B)

| | F | P |
|---|---|---|
| **Soil** | 1.27 | **0.01** |
| pH | 3.04 | **0.01** |
| $NH_4^+$ | 1.88 | **0.01** |
| Labile C | 1.22 | **0.09** |
| BD | 1.29 | **0.08** |
| TN | 0.85 | 0.80 |
| TC | 0.82 | 0.87 |
| **Plant** | 1.26 | **0.01** |
| Litter | 1.60 | **0.01** |
| LAI | 1.14 | 0.16 |
| $C_3$-PB | 0.96 | 0.53 |
| NDVI | 1.43 | **0.02** |
| BNPP | 1.69 | **0.01** |
| $C_4$-PB | 1.04 | 0.37 |
| **Tm & MS** | 1.41 | **0.02** |
| Tm | 1.81 | **0.01** |
| MS | 1.05 | 0.32 |

(C)

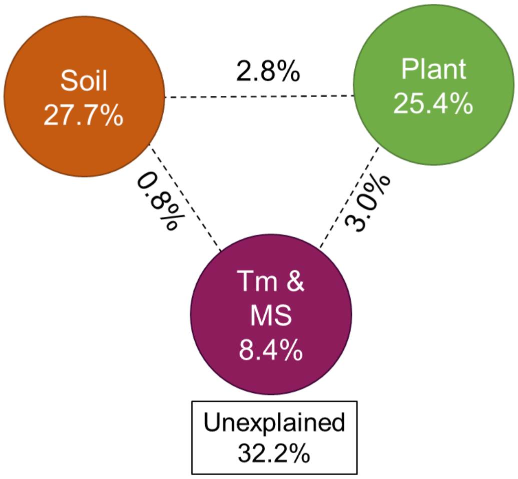


**Fig. S2.** Canonical correspondence analysis (CCA) for GeoChip data and environmental attributes. (A) CCA plot showed that microbial community functional composition was strongly shaped by key environmental factors, including soil temperature (T), moisture (M), pH, bulk

density (BD), soil total organic C (TOC), Labile C pool, total nitrogen (TN), $NH_4^+$, litter mass, $C_3$ peak biomass ($C_3$-PB), $C_4$ peak biomass ($C_4$-PB), belowground net primary productivity (BNPP), normalized difference vegetation index (NDVI) and leaf area index (LAI). (B) The inserted table showed the significance of each or subsets of the environmental variables in explaining the variations of microbial community functional gene composition based on F-test. (C) CCA-based variation partitioning analysis showed the proportions of community structure variations that can be explained by different types of environmental factors. The circles show the variation explained by each group of environmental factors alone. The numbers between the circles show the interactions of the two factors on both sides. UU stands for unclipped-unwarmed, UW for unclipped-warmed, CU for clipped-unwarmed and CW for clipped-warmed plots.
