# Supplementary material for: Annual Removal of Aboveground Plant Biomass Alters Soil Microbial Responses to Warming
Source: mBio. 2016 Sep 27;7(5):e00976-16. doi: 10.1128/mBio.00976-16 (PMC5040111; doi:10.1128/mBio.00976-16)
Supplement: Figure S4 — Detrended correspondence analysis (DCA) for microbial community composition measured by GeoChip (a); 454 sequencing of 16S rRNA gene communities (b); and PLFAs (c). Download [file mbo005163005sf4.pdf]

(A)

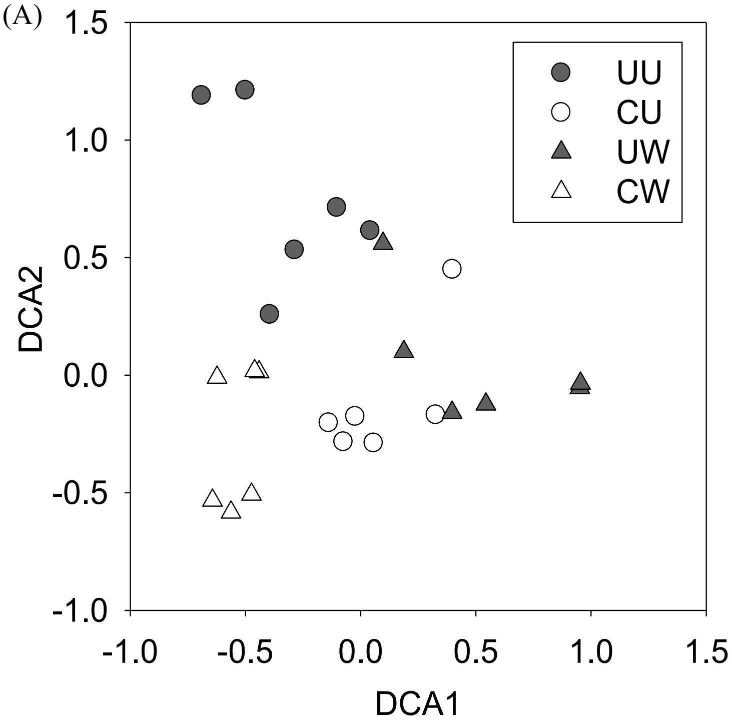

UU
CU
UW
CW
DCA2
DCA1
1.5
1.0
0.5
0.0
-0.5
-1.0
-1.0
-0.5
0.0
0.5
1.0
1.5

(B)

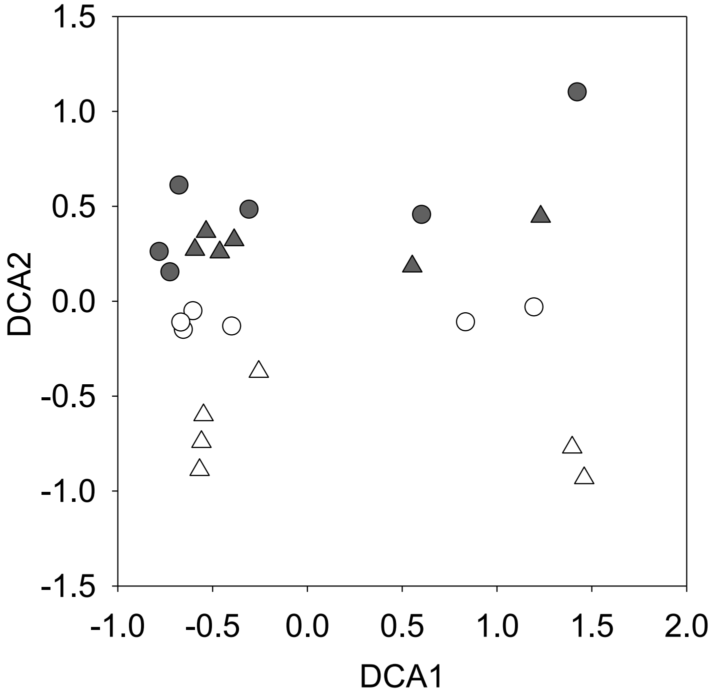

DCA2
DCA1
1.5
1.0
0.5
0.0
-0.5
-1.0
-1.5
-1.0
-0.5
0.0
0.5
1.0
1.5
2.0

(C)

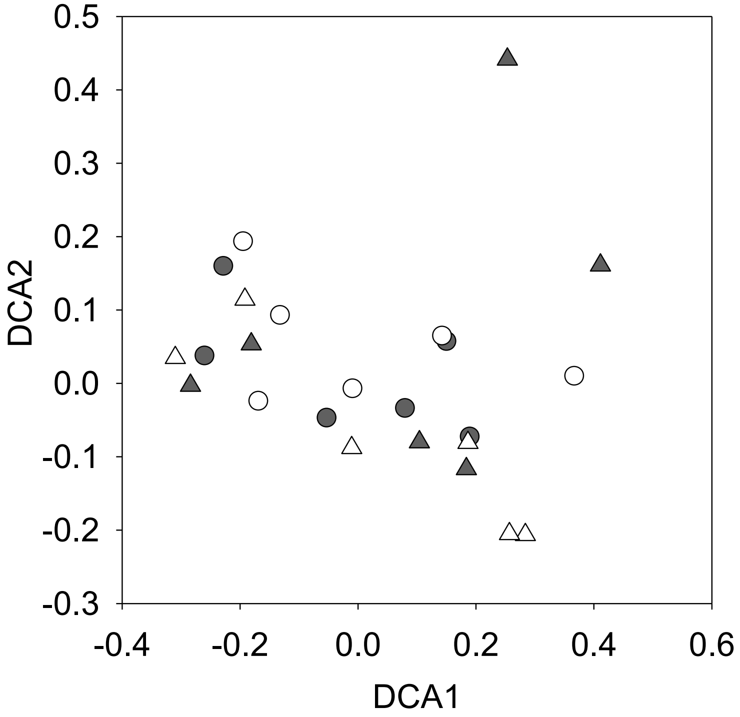

Figure S3. Detrended correspondence analysis (DCA) for microbial community composition measured by GeoChip (A); 454 sequencing of 16S rRNA gene (B); and PLFAs (C). UU stands for unclipped-unwarmed, UW for unclipped-warmed, CU for clipped-unwarmed and CW for clipped-warmed plots.
