# Supplementary material for: Annual Removal of Aboveground Plant Biomass Alters Soil Microbial Responses to Warming
Source: mBio. 2016 Sep 27;7(5):e00976-16. doi: 10.1128/mBio.00976-16 (PMC5040111; doi:10.1128/mBio.00976-16)
Supplement: Figure S5 — Changes of 16S rRNA genes at the phylum level by 454 sequencing in response to treatments. Download [file mbo005163005sf5.pdf]

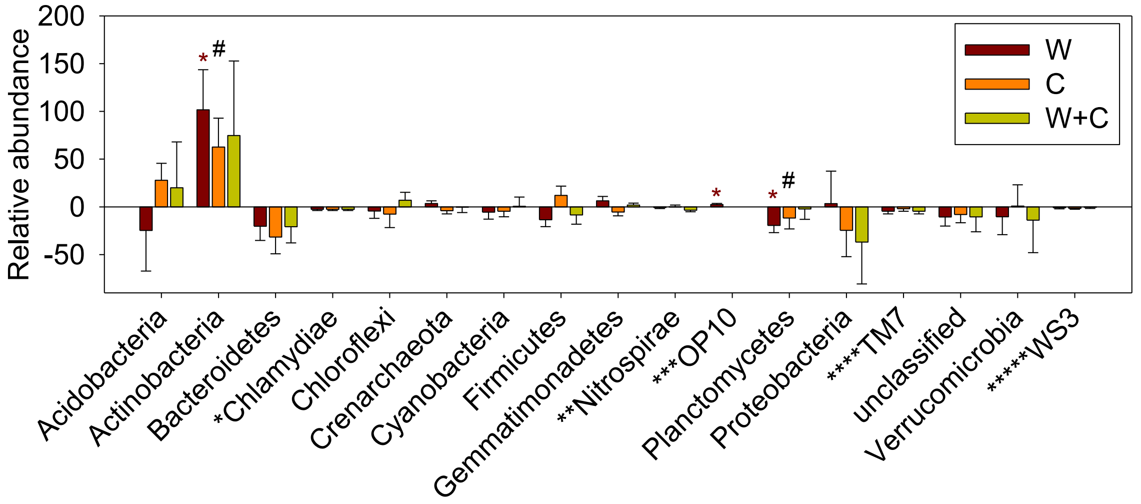

Figure S4. Changes of 16S rRNA genes at the phyla level by 454 sequencing in response to treatments. Bars presented show mean ± standard error (n = 6) of the relative abundance of the phyla in response to warming alone (W), clipping alone (C) and their combination (W+C) as compared with control (unclipped-unwarmed treatment). Significance of treatment effect is indicated by * when $p \leq 0.10$, and in the same colors as bars for different treatment groups. Significance of interaction between warming and clipping is labeled with # when $p \leq 0.10$. Interaction on Actinobacteria was antagonistic, and that on Planctomycetes was synergistic. * Chlamydiae sequences were only detected in UU treatment; ** Nitrospirae sequences were only detected in CU, UU, and UW treatments; *** OP10 sequences were only detected in UW treatment; **** TM7 sequences were only detected in CU and UU treatments; ***** WS3 sequences were only detected in UU, CW, and UW treatments.
