# Supplementary material for: Annual Removal of Aboveground Plant Biomass Alters Soil Microbial Responses to Warming
Source: mBio. 2016 Sep 27;7(5):e00976-16. doi: 10.1128/mBio.00976-16 (PMC5040111; doi:10.1128/mBio.00976-16)
Supplement: Table S1 — Soil and plant properties (means ± standard errors) in different treatments and ANOVA results. [file mbo005163005st1.docx]

**Table S1**. Soil and plant properties (mean ± standard error) in different treatments and ANOVA results. UU stands for unclipped-unwarmed, UW for unclipped-warmed, CU for clipped-unwarmed and CW for clipped-warmed plots.

| Category/Variable | | UU | UW | CU | CW | ANOVA | | | | | |
| --- | --- | --- | --- | --- | --- | --- | --- | --- | --- | --- | --- |
|  |  |  |  |  |  | warming | | clipping | | Warming:clipping | |
|  |  |  |  |  |  | F | P | F | P | F | P |
| Soil properties | Temperature (°C) | 15.01±0.13 | 16.23±0.22 | 15.14±0.10 | 17.18±0.19 | 131.88 | **<0.001** | 14.28 | **0.002** | 8.38 | **0.011** |
|  | Moisture | 30.03±0.79 | 28.64±0.58 | 29.73±0.77 | 28.70±0.55 | 14.21 | **0.002** | 0.13 | 0.720 | 0.31 | 0.587 |
|  | Bulk density | 1.28±0.05 | 1.31±0.03 | 1.40±.05 | 1.34±0.04 | 0.31 | 0.583 | 5.93 | **0.028** | 2.25 | 0.154 |
|  | Total organic C | 1.37±0.10 | 1.43±0.20 | 1.35±0.17 | 1.47±0.24 | 1.28 | 0.275 | 0.04 | 0.845 | 0.17 | 0.686 |
|  | Labile C Pool 1 | 2.77±0.42 | 2.75±0.33 | 2.57±0.37 | 2.72±0.27 | 0.06 | 0.805 | 0.19 | 0.667 | 0.14 | 0.712 |
|  | Labile C pool 2 | 3.84±0.51 | 4.34±0.58 | 3.76±0.43 | 4.35±0.64 | 3.01 | 0.103 | 0.01 | 0.912 | 0.05 | 0.826 |
|  | Recalcitrant C pool | 7.09±0.75 | 7.17±1.62 | 7.22±1.33 | 7.66±1.92 | 0.14 | 0.717 | 0.19 | 0.670 | 0.07 | 0.801 |
|  | C derived from C_4_ plant | 0.60±0.04 | 0.71±0.05 | 0.63±0.05 | 0.74±0.03 | 12.76 | **0.003** | 0.93 | 0.350 | 0.08 | 0.776 |
|  | Total N | 0.11±0.01 | 0.12±0.01 | 0.11±0.01 | 0.12±0.01 | 0.93 | 0.350 | 0.004 | 0.950 | 0.70 | 0.416 |
|  | NH_4_^+^ | 1.44±0.14 | 1.35±0.13 | 1.16±0.12 | 1.46±0.14 | 2.79 | 0.116 | 1.83 | 0.197 | 10.01 | **0.006** |
|  | NO_3_^-^ | 0.63±0.29 | 0.48±0.18 | 0.44±0.12 | 0.41±0.13 | 0.86 | 0.368 | 2.02 | 0.176 | 0.47 | 0.503 |
|  | C/N ratio | 11.95±0.39 | 12.28±1.11 | 12.13±0.60 | 11.99±0.75 | 0.06 | 0.814 | 0.02 | 0.890 | 0.31 | 0.584 |
|  | ^13^C | -18.48±0.53 | -17.26±0.76 | -18.08±0.79 | -16.96±0.47 | 7.17 | **0.017** | 0.96 | 0.342 | 0.01 | 0.930 |
|  | ^15^N | 2.66±0.30 | 3.09±0.21 | 2.57±0.24 | 3.88±0.10 | 40.85 | **<0.001** | 6.09 | **0.026** | 9.17 | **0.008** |
|  | Phenol oxidase | 4.09±0.57 | 4.23±0.58 | 4.75±0.69 | 5.71±2.14 | 0.20 | 0.655 | 3.36 | **0.073** | 0.16 | 0.363 |
| Soil respiration | Total | 2.04±0.16 | 2.64±0.23 | 2.45±0.11 | 3.12±0.26 | 14.12 | **0.002** | 6.95 | **0.019** | 0.05 | 0.826 |
| PLFAs | Total PLFAs | 136.15±15.70 | 183.68±10.57 | 159.38±20.13 | 211.67±14.21 | 17.34 | **0.001** | 3.16 | **0.097** | 0.06 | 0.817 |
|  | Fungi/Bacteria | 0.57±0.07 | 0.53±0.05 | 0.65±0.11 | 0.57±0.07 | 0.83 | 0.378 | 0.58 | 0.460 | 0.16 | 0.692 |
| Plant | C_3_ plant peak biomass | 58.25±39.72 | 78.14±2.95 | 78.73±28.93 | 87.70±15.35 | 0.56 | 0.464 | 0.61 | 0.446 | 0.08 | 0.780 |
|  | C_4_ plant peak biomass | 174.69±4.64 | 218.45±11.56 | 177.09±14.82 | 255.98±45.87 | 8.14 | **0.012** | 0.86 | 0.368 | 0.67 | 0.427 |
|  | BNPP | 335.35±39.85 | 410.61±29.11 | 370.28±39.57 | 702.68±34.22 | 27.83 | **<0.001** | 17.91 | **0.001** | 11.07 | **0.005** |
|  | Litter | 655.88±86.82 | 838.40±92.84 | 125.93±14.76 | 162.18±23.53 | 4.46 | **0.052** | 135.51 | **<0.001** | 1.99 | 0.178 |

Bold values represent the significance at p < 0.10. The directions of changes are presented in Table 1.
