# Supplementary material for: Annual Removal of Aboveground Plant Biomass Alters Soil Microbial Responses to Warming
Source: mBio. 2016 Sep 27;7(5):e00976-16. doi: 10.1128/mBio.00976-16 (PMC5040111; doi:10.1128/mBio.00976-16)
Supplement: Table S2 — Interactive effects of warming and clipping on carbon degradation and nitrogen cycling genes measured by GeoChip. [file mbo005163005st2.docx]

**Table S2**. Interactive effects of warming and clipping on carbon degradation and nitrogen cycling genes measured by GeoChip.

| Functional category | | Genes | OE^a^ (%) | PE^b^ (%) | OE-PE | Interactive effect^c^ |
| --- | --- | --- | --- | --- | --- | --- |
| C degradation | Starch  degradation | *amyA* | 82.13 | 32.90 | **-49.23**** | **Antagonistic** |
|  |  | *amyX* | -102.37 | -26.70 | 75.67 | Additive |
|  |  | glucoamylase | 33.69 | 40.78 | 7.09 | Additive |
|  |  | *pulA* | -6.58 | 6.03 | 12.61 | Additive |
|  | Hemi-cellulose  degradation | *ara* | 501.19 | 199.02 | **-302.17***** | **Antagonistic** |
|  |  | *ara*_fungi | 199.82 | 45.03 | **-154.79**** | **Antagonistic** |
|  |  | *xylA* | -37.20 | -39.25 | -2.05 | Additive |
|  |  | xylanase | 41.72 | 4.15 | -37.57 | Additive |
|  | Cellulose  degradation | CDH | 117.00 | 70.37 | -46.63 | Additive |
|  |  | cellobiase | 67.47 | 37.47 | -30.00 | Additive |
|  |  | endoglucanase | -25.78 | 61.71 | 87.49 | Additive |
|  |  | exoglucanase | 110.05 | -24.65 | -134.70 | Additive |
|  | Chitin  degradation | acetyl-glucosaminidase | 193.18 | 103.00 | **-90.18*** | **Antagonistic** |
|  |  | endochitinase | 119.31 | 75.21 | -44.10 | Additive |
|  |  | exochitinase | 55.39 | 73.42 | 18.03 | Additive |
|  | Pectin  degradation | pectinase | -9.58 | 57.28 | 66.86 | Additive |
|  | Aromatics  degradation | *limEH* | 78.01 | 136.56 | 58.55 | Additive |
|  |  | *vanA* | 10.58 | 17.80 | 7.22 | Additive |
|  |  | *vdh* | 198.00 | 99.74 | -98.26 | Additive |
|  | Lignin  degradation | *glx* | -17.40 | 8.86 | 26.26 | Additive |
|  |  | *lip* | 15.74 | 38.23 | 22.49 | Additive |
|  |  | *mnp* | 73.21 | 38.08 | -35.13 | Additive |
|  |  | phenol_oxidase | 37.58 | 24.49 | -13.09 | Additive |
| N cycling | Dissimilatory N reduction | *napA* | 71.40 | 155.02 | -83.62 | Additive |
|  |  | *nrfA* | -7.18 | 13.89 | -21.07 | Additive |
|  | Assimilatory N reduction | *nasA* | 66.99 | 118.86 | -51.87 | Additive |
|  | Denitrification | *narG* | 6.45 | 7.73 | -1.28 | Additive |
|  |  | *nirK* | 27.58 | 97.71 | **-70.13***** | **Antagonistic** |
|  |  | *nirS* | 16.88 | 47.30 | -30.42 | Additive |
|  |  | *norB* | 39.76 | 67.72 | -27.96 | Additive |
|  |  | *nosZ* | 50.64 | 112.47 | **-61.83**** | **Antagonistic** |
|  | Ammonification | *gdh*^d^ | na | na | na | Additive |
|  |  | *ureC* | 31.48 | 50.83 | -19.35 | Additive |
|  | Nitrogen fixation | *nifH* | 27.23 | 55.05 | **-27.82**** | **Antagonistic** |

The significance of contrasts by two tailed paired t test are in bold font and labeled as “***” when p ≤ 0.01, “**” when 0.01 <p ≤ 0.05.

^a^OE: observed effect. Calculated as 100% × (CW - UU) / UU, where CW and UU represent the averaged gene signal intensities in clipped-warmed and unclipped-unwarmed plots, respectively;

^b^PE: predicted additive effect. Calculated as [100% × (UW – UU)/UU + 100% × (CU - UU)/UU], where UW and CU represent averaged gene signal intensities in unclipped-warmed and clipped-unwarmed plots, respectively;

^c^Interactive effect is additive when PE does not differ significantly from OE, synergistic when PE is significantly smaller than OE, or antagonistic when PE is significantly larger than OE;

^d^For *gdh* gene, no probe showed positive signal in unclipped-unwarmed plots, so UU = 0;
