# Supplementary material for: Annual Removal of Aboveground Plant Biomass Alters Soil Microbial Responses to Warming
Source: mBio. 2016 Sep 27;7(5):e00976-16. doi: 10.1128/mBio.00976-16 (PMC5040111; doi:10.1128/mBio.00976-16)
Supplement: Table S3 — Percent changes of carbon degradation and nitrogen cycling genes measured by GeoChip in response to warming in unclipped and clipped plots or in response to clipping in unwarmed and warmed plots. [file mbo005163005st3.docx]

**Table 3**. Percent changes of carbon degradation and nitrogen cycling genes measured by GeoChip in response to warming in unclipped and clipped plots, or to clipping in unwarmed and warmed plots.

| Functional category | | Genes | Probe # | Warming effect (%) | | Clipping effect (%) | |
| --- | --- | --- | --- | --- | --- | --- | --- |
|  |  |  |  | UW Vs UU^a^ | CW Vs CU^b^ | CU Vs UU^c^ | CW Vs UW^d^ |
| C degradation | Starch  degradation | *amyA* | 44 | **51.09***** | 1.42 | **31.04**** | -12.04 |
|  |  | *amyX* | 1 | -49.14 | 56.72 | -53.23 | 44.14 |
|  |  | glucoamylase | 7 | **64.58**** | **103.70**** | -30.89 | -14.46 |
|  |  | *pulA* | 11 | 5.93 | 21.19 | -12.51 | 0.09 |
|  | Hemi-cellulose  degradation | *ara* | 15 | **163.66**** | -31.66 | **337.53***** | 13.41 |
|  |  | *ara*_fungi | 9 | **91.40*** | -30.41 | **108.42**** | -24.23 |
|  |  | *xylA* | 10 | -17.59 | -24.44 | -19.61 | -26.28 |
|  |  | xylanase | 15 | **36.74*** | -0.80 | 4.98 | -23.84 |
|  | Cellulose  degradation | CDH | 8 | **67.18**** | 13.72 | **49.82*** | 1.91 |
|  |  | cellobiase | 13 | 25.44 | -3.21 | **42.03*** | 9.59 |
|  |  | endoglucanase | 4 | 24.37 | **224.40*** | -50.15 | 30.02 |
|  |  | exoglucanase | 3 | 27.08 | -58.82 | 82.97 | -40.70 |
|  | Chitin  degradation | acetyl-glucosaminidase | 6 | **112.51***** | 12.36 | **80.67***** | -4.47 |
|  |  | endochitinase | 21 | **69.07***** | 16.62 | **50.24**** | 3.63 |
|  |  | exochitinase | 3 | 17.78 | 26.02 | 37.61 | 47.24 |
|  | Pectin  degradation | pectinase | 4 | -19.34 | 43.30 | 9.76 | **95.00**** |
|  | Aromatics  degradation | *limEH* | 6 | 25.81 | **55.43*** | 52.20 | **88.03**** |
|  |  | *vanA* | 20 | 9.14 | 16.13 | 1.44 | 7.94 |
|  |  | *vdh* | 4 | 75.97 | -10.04 | **122.03**** | 13.51 |
|  | Lignin  degradation | *glx* | 4 | -56.10 | -21.51 | 38.70 | 147.98 |
|  |  | *lip* | 7 | 22.41 | **48.11**** | -6.67 | 12.93 |
|  |  | *mnp* | 8 | 26.17 | -6.09 | **47.04*** | 9.44 |
|  |  | phenol_oxidase | 18 | -16.34 | -19.12 | **53.92**** | 48.81 |
| N cycling | Dissimilatory N reduction | *napA* | 11 | **115.68**** | 23.01 | 39.34 | -20.53 |
|  |  | *nrfA* | 12 | 7.42 | -12.82 | 6.47 | -13.59 |
|  | Assimilatory N reduction | *nasA* | 15 | **76.21**** | 17.06 | 42.65 | -5.23 |
|  | Denitrification | *narG* | 51 | 8.02 | 6.77 | -0.29 | -1.45 |
|  |  | *nirK* | 43 | **66.15***** | -3.03 | **31.56**** | **-23.21**** |
|  |  | *nirS* | 34 | **41.11**** | 10.06 | 6.19 | -17.17 |
|  |  | *norB* | 15 | **64.60**** | 35.53 | 3.12 | -15.09 |
|  |  | *nosZ* | 39 | **73.64***** | 8.51 | **38.83**** | -13.24 |
|  | Ammonification | *gdh*^e^ | 3 | na | -8.78 | na | -3.01 |
|  |  | *ureC* | 38 | 14.84 | -3.32 | **35.99**** | 14.49 |
|  | Nitrogen fixation | *nifH* | 83 | **23.17**** | -3.52 | **31.88***** | 3.30 |

The significance of the comparison by two tailed paired t test is labeled as “***” when p ≤ 0.01, “**” when 0.01 < p ≤ 0.05, and “*” when 0.05 < p ≤ 0.10;

^a^UW Vs UU: 100% × (UW - UU) / UU, where UW and UU represent the averaged gene signal intensities in unclipped-warmed and unclipped-unwarmed plots, respectively;

^b^CW Vs CU: 100% × (CW - CU) / CU, where CW and CU represent averaged gene signal intensities in clipped-warmed and clipped-unwarmed plots, respectively;

^c^CU Vs UU: 100% × (CU - UU) / UU;

^d^CW Vs UW: 100% × (CW - UW) / UW;

^e^For *gdh* gene, no probe showed positive signal in unclipped-unwarmed plots, so UU = 0.
