# Supplementary material for: Annual Removal of Aboveground Plant Biomass Alters Soil Microbial Responses to Warming
Source: mBio. 2016 Sep 27;7(5):e00976-16. doi: 10.1128/mBio.00976-16 (PMC5040111; doi:10.1128/mBio.00976-16)
Supplement: Table S4 — Changes of alpha diversity in response to treatments compared with the control results for functional gene compositions detected by GeoChip and for taxonomic compositions analyzed by 454 sequencing of 16S rRNA genes. [file mbo005163005st4.docx]

**Table 4**. Changes of alpha diversity in response to treatments as compared with control (UU) for functional gene compositions detected by GeoChip, and for taxonomic compositions analyzed by 454 sequencing of 16S rRNA genes.

| Index | GeoChip | | | 16S | | |
| --- | --- | --- | --- | --- | --- | --- |
|  | Warming alone^a^ | Clipping alone^b^ | Combined effect^c^ | Warming alone^a^ | Clipping alone^b^ | Combined effect^c^ |
| Richness | 271±163 | *** 196±89** | 220±267 | 41±33 | -57±63 | -89±71 |
| Evenness | 0.00002±0.00003 | -0.00002±0.00001 | 0±0.00004 | -0.0022±0.0053 | -0.0052±0.005 | 0.0023±0.0056 |
| Shannon | 0.33±0.25 | *** 0.34±0.16** | 0.28±0.25 | 0.06±0.05 | -0.15±0.08 | -0.14±0.11 |
| Simpson | 0.0005±0.0005 | *** 0.0006±0.0003** | 0.0005±0.0003 | 0.0002±0.0005 | -0.0011±0.0004 | -0.0005±0.0006 |
| Inverse Simpson | 269±161 | *** 195±88** | 219±265 | 3±21 | *** -56±18** | -43±22 |

*Bold values represent the significance at p ≤ 0.10.

^a^Warming effect is calculated as UW-UU, where UW and UU stand for the diversity index in unclipped-warmed, and unclipped-unwarmed plots, respectively.

^b^Clipping effect is calculated as CU-UU, where CU stands for the diversity index in clipped-unwarmed plots.

^c^Combined effect is calculated as CW-UU, where CW stands for the diversity index in clipped-warmed plots.
