# Supplementary material for: Annual Removal of Aboveground Plant Biomass Alters Soil Microbial Responses to Warming
Source: mBio. 2016 Sep 27;7(5):e00976-16. doi: 10.1128/mBio.00976-16 (PMC5040111; doi:10.1128/mBio.00976-16)
Supplement: Table S5 — Mantel tests for comparisons between individual C degradation and nitrogen cycling functional gene compositions based on GeoChip and the soil and plant property variables. [file mbo005163005st5.docx]

**Table S5**. Mantel tests between C degradation and N cycling functional gene compositions based on GeoChip and the environmental variables. The environmental variables include temperature (T), moisture (M), bulk density (BD), total organic C (TOC), labile C pool 1 (LP1), labile C pool 2 (LP2), recalcitrant C pool (RP), C derived from C_4_ plant (F-C_4_), total N (TN), NH_4_^+^, NO_3_^-^, C/N ratio (C/N), ^13^C and ^15^N in soil; CO_2_ flux (CO_2_) and nitrogen mineralization rate (NM); C_3_ peak biomass (C_3_-PB), C_4_ peak biomass (C_4_-PB), belowground net primary productivity (BNPP) and litter mass.

| Gene/Category | T | M | BD | TOC | LP1 | LP2 | RP | F-C_4_ | TN | NH_4_^+^ | NO_3_^-^ | C/N | ^13^C | ^15^N | CO_2_ | NM | C_3_-PB | C_4_-PB | BNPP | Litter |
| --- | --- | --- | --- | --- | --- | --- | --- | --- | --- | --- | --- | --- | --- | --- | --- | --- | --- | --- | --- | --- |
| *amyA* |  |  |  |  |  |  |  |  |  |  |  |  |  |  |  |  |  | ** |  | ** |
| *amyX*^a^ |  |  |  |  |  |  |  |  |  |  |  |  |  |  |  |  |  |  |  |  |
| glucoamylase | ** |  |  |  |  |  |  |  |  |  |  |  |  |  |  |  |  |  | ** | ** |
| *pulA* |  |  |  |  |  |  |  |  |  |  |  |  |  |  | ** |  |  |  | ** |  |
| *ara* |  |  |  |  |  |  |  |  |  |  |  |  |  |  |  |  |  | ** |  |  |
| *ara*_fungi |  |  |  |  |  |  |  |  |  |  |  |  |  |  |  |  |  |  |  |  |
| *xylA* |  | ** |  |  |  |  |  |  |  |  |  |  |  |  |  |  |  |  |  |  |
| *xylanase* |  |  |  |  |  |  |  |  |  | ** |  |  |  |  |  |  |  |  |  |  |
| CDH |  |  |  |  | ** |  |  |  |  |  |  |  |  |  |  |  |  |  |  |  |
| cellobiase |  |  |  |  |  |  |  | ** |  |  |  |  | ** |  |  |  |  |  |  |  |
| endoglucanase | ** |  |  | ** |  |  | ** |  |  | ** |  |  |  |  | ** |  |  | ** |  | ** |
| exoglucanase |  |  |  |  |  |  |  |  |  |  |  |  |  |  |  |  |  |  |  |  |
| acetylglucosaminidase |  |  |  |  |  |  |  |  |  |  |  |  |  |  |  | *** |  |  |  | *** |
| endochitinase | *** |  |  |  |  |  |  |  |  |  |  |  |  |  |  |  |  |  | ** |  |
| exochitinase |  |  |  |  |  | ** |  |  |  | ** |  |  |  |  |  |  |  |  |  | ** |
| pectinase | ** |  |  |  |  |  |  |  |  |  |  |  |  |  |  |  |  |  |  |  |
| *limEH* | ** |  |  |  |  |  |  |  |  |  |  |  |  |  |  |  |  |  | ** |  |
| *vanA* |  |  |  |  |  |  |  |  |  |  |  |  |  |  |  |  | ** |  |  |  |
| *vdh* |  |  |  |  |  |  |  |  |  |  |  |  |  |  |  |  |  |  |  |  |
| **Non-lignin** | ****** |  |  |  |  |  | ****** |  |  |  |  |  | ****** |  |  |  |  | ****** |  | ****** |
| *glx* |  |  |  | ** |  |  | *** |  | ** |  |  |  |  |  |  |  |  |  |  |  |
| *lip* |  |  |  | ** |  |  | ** |  |  | ** | ** |  |  |  | ** |  | ** |  |  |  |
| *mnp* |  |  |  |  |  |  |  |  |  | ** |  |  |  |  | ** | ** |  | *** |  |  |
| phenol_oxidase | ** |  |  |  |  |  |  |  |  |  |  |  |  |  |  |  |  |  | ** |  |
| **Lignin** | ****** |  |  | ****** |  |  | ****** |  | ****** | ****** |  |  |  |  |  |  |  |  | ****** |  |
| **All C degradation genes** | ****** |  |  |  |  |  | ****** |  |  |  |  |  |  |  |  |  |  | ****** | ****** | ****** |
| Gene/Category | T | M | BD | TOC | LP1 | LP2 | RP | F-C_4_ | TN | NH_4_^+^ | NO_3_^-^ | C/N | ^13^C | ^15^N | CO_2_ | NM | C_3_-PB | C_4_-PB | BNPP | Litter |
| *napA* |  |  |  |  |  |  | ** |  |  |  |  |  |  |  | ** |  |  |  |  | *** |
| *nrfA* |  |  | ** |  |  |  |  | ** |  |  |  |  |  |  |  |  |  |  |  |  |
| **Dissimilatory N reduction** |  |  |  |  |  |  |  |  |  |  |  |  |  |  |  |  |  |  |  | ****** |
| *nasA* | ** |  |  |  |  |  |  |  |  | ** |  | ** |  |  |  |  |  |  |  |  |
| *narG* |  |  |  | ** |  |  | ** |  |  | *** |  |  |  |  |  |  |  |  |  |  |
| *nirK* |  |  |  |  |  |  |  |  |  |  |  |  |  |  |  |  |  |  |  |  |
| *nirS* |  |  |  |  |  |  |  |  |  | ** |  |  |  |  |  |  |  |  |  |  |
| *norB* | ** |  |  |  | ** |  |  |  |  |  |  |  |  |  |  |  |  |  |  |  |
| *nosZ* | ** |  |  |  |  |  |  |  |  |  |  |  |  |  |  |  |  |  |  |  |
| **Denitrification** |  |  |  |  |  |  |  |  |  | ****** |  |  |  |  |  |  |  |  |  | ****** |
| *gdh* |  |  |  | ** |  |  | ** |  | ** |  |  |  | ** |  |  |  |  | ** |  |  |
| *ureC* |  |  |  |  |  |  | ** |  |  |  |  |  |  |  |  |  |  |  |  |  |
| **Ammonification** |  |  |  | ****** |  |  | ****** |  |  |  |  |  |  |  |  |  |  |  |  |  |
| *nifH* |  |  |  |  |  |  |  |  |  | ** |  |  |  |  |  |  |  |  |  |  |
| **All N cycling genes** |  |  |  |  |  |  |  |  |  | ****** |  |  |  |  |  |  |  |  |  |  |

Significance is indicated with “***” when p<0.01 and “**” when p<0.05. Results for categories containing more than one genes are in red. Results for categories containing more than one genes are in red and bold font.

^a^Only one probe was detected in *amyX* gene and Pearson correlation tests were performed between *amyX* and the environmental variables.
